# Qualitative analysis of visual risk communication on twitter during the Covid-19 pandemic

**DOI:** 10.1186/s12889-021-10851-4

**Published:** 2021-04-28

**Authors:** Joanna Sleigh, Julia Amann, Manuel Schneider, Effy Vayena

**Affiliations:** Department of Health Science and Technology, ETH, Zürich, Switzerland

**Keywords:** Twitter, Public health, Risk communication, Visuals, Pandemic, Covid-19

## Abstract

**Background:**

The Covid-19 pandemic is characterized by uncertainty and constant change, forcing governments and health authorities to ramp up risk communication efforts. Consequently, visuality and social media platforms like Twitter have come to play a vital role in disseminating prevention messages widely. Yet to date, only little is known about what characterizes visual risk communication during the Covid-19 pandemic. To address this gap in the literature, this study’s objective was to determine how visual risk communication was used on Twitter to promote the World Health Organisations (WHO) recommended preventative behaviours and how this communication changed over time.

**Methods:**

We sourced Twitter’s 500 most retweeted Covid-19 messages for each month from January–October 2020 using Crowdbreaks. For inclusion, tweets had to have visuals, be in English, come from verified accounts, and contain one of the keywords ‘covid19’, ‘coronavirus’, ‘corona’, or ‘covid’. Following a retrospective approach, we then performed a qualitative content analysis of the 616 tweets meeting inclusion criteria.

**Results:**

Our results show communication dynamics changed over the course of the pandemic. At the start, most retweeted preventative messages came from the media and health and government institutions, but overall, personal accounts with many followers (51.3%) predominated, and their tweets had the highest spread (10.0%, i.e., retweet count divided by followers). Messages used mostly photographs and images were found to be rich with information. 78.1% of Tweets contained 1–2 preventative messages, whereby ‘stay home’ and ‘wear a mask’ frequented most. Although more tweets used health loss framing, health gain messages spread more.

**Conclusion:**

Our findings can inform the didactics of future crisis communication. The results underscore the value of engaging individuals, particularly influencers, as advocates to spread health risk messages and promote solidarity. Further, our findings on the visual characteristic of the most retweeted tweets highlight factors that health and government organisations should consider when creating visual health messages for Twitter. However, that more tweets used the emotive medium of photographs often combined with health loss framing raises concerns about persuasive tactics. More research is needed to understand the implications of framing and its impact on public perceptions and behaviours.

**Supplementary Information:**

The online version contains supplementary material available at 10.1186/s12889-021-10851-4.

## Background

The coronavirus pandemic has offered public health professionals a real-world case study on visual risk communication through social media platforms like Twitter. In the wake of the pandemic, amid toilet-paper buying frenzies, government-ordered city shutdowns, and requests from mayors that citizens stay home, visual health risk messages have proliferated online. From health officials and pop-stars co-producing YouTube videos [1, 2] to animals explaining health measures on TikTok [3], health risk communication has become fully submerged in the image-driven, de-centralised, peer-to-peer, cross-cultural melting pot of social media. Among the array of social media platforms, Twitter has played a prominent role by allowing everyone, from public officials to citizens, to easily share and consume visual and multimedia infection prevention and control messages.

In online environments, such as Twitter, visuality can help to communicate health and risk messages. Graphics (when accurate and truthful) can improve public understanding of qualitative and quantitative health risk information [4]. In doing so, they then foster autonomy by enabling viewers to make their own health decisions and facilitating shared-medical decisions and behaviour change [5, 6]. Visuals can also help to engage ‘hard to reach’ audiences, such as those with low literacy levels, thereby promoting social equity as an ethical imperative of public health [7]. Moreover, visuals can prompt action by their persuasive and emotional impact [8–10], with colour hues affecting an individual’s psychological reactance to health recommendations [11]. Ultimately, their ability to affect viewers renders them powerful tools to foster public adoption of health officials’ recommendations [12]. When new infectious diseases break out, visuals can help risk-reducing messages stand out in the seas of information on social media. In this way, they can help reach and be understood by a majority of the population, promoting solidarity and reducing stigmatisation of risk groups [13].

As one of the most used and well-established social media platforms, with a history of use during public emergencies, natural disasters and epidemics, Twitter holds great potential for strategic and cost-effective visual health risk communication. Not only as it allows public health authorities and government agencies to reach millions of people and communities, but Twitter also enables research, monitoring and evaluation of health communication campaigns. However, like all social media channels, along with this vast potential, Twitter faces corresponding challenges and ethical concerns [14]. With this platform, the lay public join journalists and topic experts as mass media and content producers, and with few filtering mechanisms, content goes viral at accelerated speeds [15, 16]. This can result in the nearly instantaneous spread of unverifiable health information, as occurred during the Ebola and Zika outbreaks [12], and now during the Covid-19 pandemic [17]. Detecting health misinformation and acting to stop its spread is a critical challenge for public health authorities, as hazardous or misleading recommendations (like drinking bleach) place individual health at risk. Content may also misrepresent statistics, thereby failing in truthfulness, sincerity, and correctness, which can lead to misunderstanding and erode trust [18, 19]. Moreover, such content saturation may promote dubious moral communication strategies, such as using shock tactics to attract attention or the sacrifice of privacy through the graphic portrayal of an individual’s story [20].

Despite the undeniable role of visual communication on social media, to date, only little is known about the characteristics of visual risk communication on Twitter during the Covid-19 pandemic. Recognizing the importance of Twitter and visual communication in a global health crisis, this study investigates the characteristics of Covid-19 risk communication visuals posted on the platform between January and October 2020. Focusing on the tweets with the most retweets for each month, we follow a retrospective approach to study tweets with visuals that contain Covid-19 prevention messages. This study’s overall objective was to determine how visual communication was used on Twitter to promote the World Health Organisations (WHO) recommended preventative behaviours and how this communication changed over time. To this end, we use qualitative content analysis [21] to identify:

1. To what extent were health and government organisations present amongst the most retweeted tweets;
2. What were the predominant graphic types and visual properties used [22];
3. Which Covid-19 preventative measures featured the most [23];
4. How health gain or loss framing was present and whether tone changed over time [24].

By providing empirical data on these four aspects of visual health communication, this study makes a timely contribution to health communication and public health research and holds important implications for practice. Specifically, by determining whose health risk messages were retweeted most often and what form these took, this study provides valuable insights into current practices of visual risk communication on Twitter. Furthermore, it pinpoints some of the potential ethical issues that different types of messages and formats raise, thereby drawing attention to the ethics of visual risk communication, a topic which has received comparatively little attention (outside discussions of fake news) during the ongoing Covid-19 pandemic.

## Methods

### Data collection

#### Data extraction

Using the platform Crowdbreaks,^1^ we sourced the tweet IDs of the most retweeted tweets (based on retweet counts at the time of request) that contained at least one of the keywords ‘covid19’, ‘coronavirus’, ‘corona’, and ‘covid’ [25]. The tweet objects (such as tweet text, publishing date, media URLs) were then received using the tweet IDs from the Twitter-API. We selected the 500 most retweeted tweets with visuals per month to see trends over time and ensure uniform distribution. The total dataset consisted of 5031 tweets. Where no tweet location was available as meta-data from Twitter, we added it manually when possible using content-based identification [26].

#### Inclusion and exclusion criteria

To be included in our analysis, tweets needed to a) include a visual (image or video), b) be in English (both image and tweet text), and c) promote a WHO recommended Covid-19 health preventative measure (as grouped in Table 1). By limiting our scope to the WHO publicised recommendations, as an internationally recognised authoritative source of Covid-19 risk communication, we sought to focus on the main preventative measures relevant to all countries. We excluded tweets that did not primarily focus on preventative Covid-19 measures, or that promoted alternative, non-WHO recommended preventative measures; for example, drinking bleach. Ambiguous cases were discussed within the research team to determine inclusion within the final sample. Upon applying these criteria, we included 616 tweets in our analysis.

**Table 1:** Codebook used for qualitative analysis

Top-level	Detail	Example
Source (Identified inductively)	Health or governmental organisation	The WHO, or Victoria Government
	Private sector	Pharma Company
	Media	CNN, ABC News
	Individual person	Citizen, politician, academic, or artist
	Other	University
Graphic Type (Saunders, 1994)	Symbols	A pictographic or logo
	Graphs	Used to show quantitative relationships
	Diagrams	Parts, a process, a general scheme, and/or the flow of results
	Illustrations or rendered pictures	Drawn pictures, realistic or abstract, including background illustrations
	Photographs	Still (i.e. photograph) or moving (such as gif or video)
	Models	Such as 3d renderings or computer models
	Composite graphics	Multiple images
Other Visual Attributes	Colour	Anything with more than white and black
	Animated	Video, Gif or animation
Link	Link / URL	A URL is in the tweet text or in the visual
Content Focus (Identified inductively)	Raises criticism	Government or political criticism, or criticism of someone’s behaviour
	Provides entertainment	Shows something funny, or emotive
	Thankful / gratitude	Thanks doctors for saving patients
Covid-19 Focus (Identified inductively)	Detection	Relates measures to detection of cases or how it impacts the body
	Treatment	Mentions people recovering
	Impact	Discusses impacts to behaviour, the economy, or society
	Other	How it spreads
Type of Action (WHO guidelines)	Social distance	Keeping distance with people and avoiding crowded places
	Wear a mask	Protecting yourself and other by wearing a mask
	Stay home	Working, studying or remaining at home if feeling unwell / quarantine
	Wash hands	Regularly and thoroughly washing hands with soap and water
	Cover mouth & nose when sneezing	Or using a tissue and disposing it immediately
	Avoid touching mouth and eyes	Particularly with unwashed hands
	Get medical help w. symptoms	(but call - don’t go in)
	Other	Cooking meat or eggs / basic hygiene / know the symptoms / get tested
Framing (Tversky & Kahneman, 1992)	Health gain	We need to *protect* ourselves and others to protect / *save* society
	Health loss	We need to follow measures to avoid sickness, suffering and death
	Non-applicable	We just need to do this

#### Qualitative content analysis

**Fig. 1:**
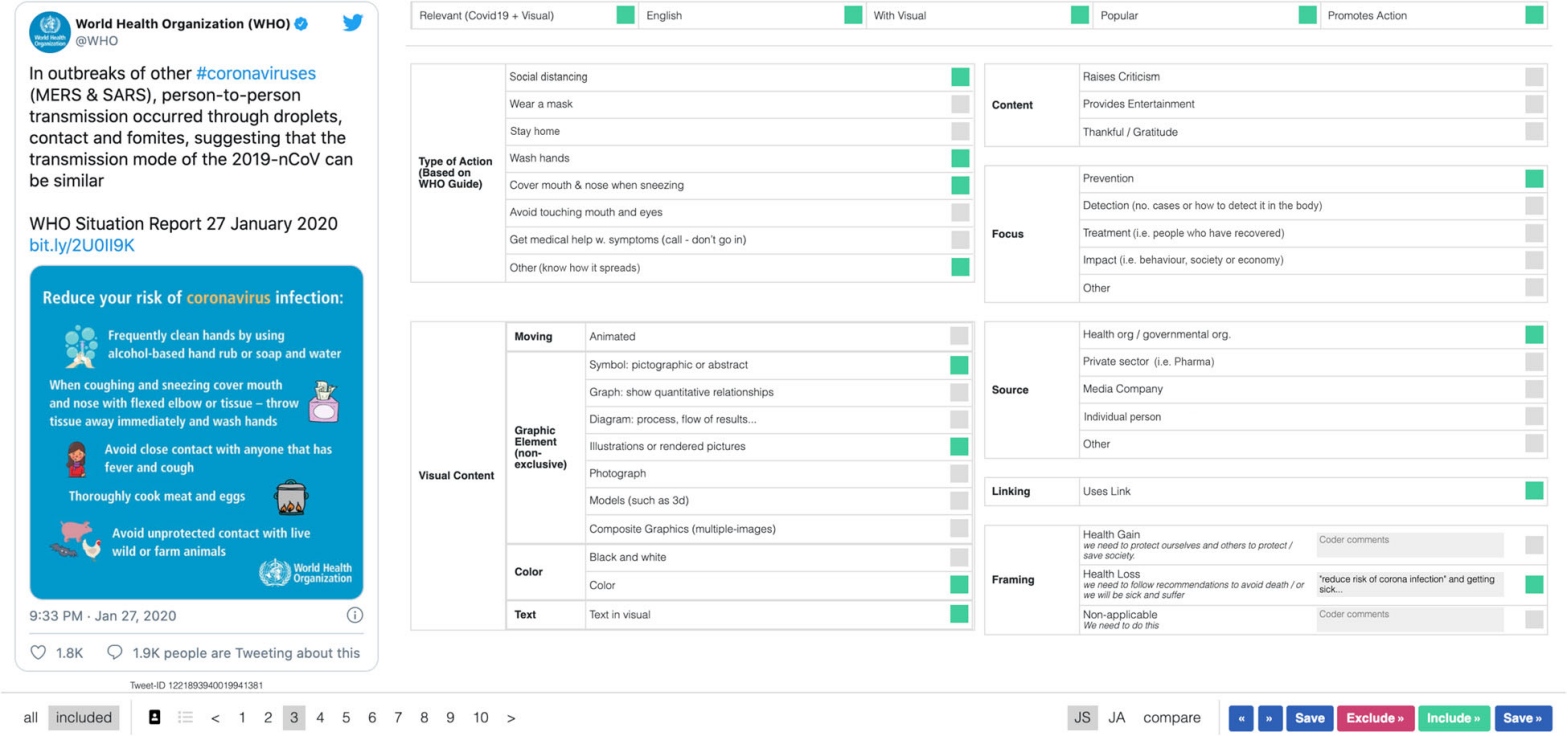
Custom Interface used for qualitative coding

We performed a qualitative content analysis following an iterative process [21]. Starting from a preliminary codebook informed by prior research and typologies [22, 24] and WHO guidelines [23], two researchers (JS, JA) coded a random sample of 40 tweets not pertaining to the study sample to test and refine the codebook. The revised codebook was then applied to a subset of 60 tweets by two researchers (JS, JA) independently to establish intercoder reliability [27, 28]. The second round of coding resulted in minor revisions to the codebook using review and discussion. One researcher (JS) then applied the final version of the codebook (Table 1) to the 616 tweets that met our inclusion criteria using a custom interface shown in Fig. 1. This interface allowed us to see the original tweet directly in the coding interface through the Twitter Embed API. A second researcher (JA) performed an intermittent reliability check on 10% of the tweets (*n* = 62) halfway through coding. Group discussions among three researchers (JS, JA, MS) resolved intermittent coding disagreements. We then analysed how the retweets were distributed for each theme and how theme items spread. The former provided insight as to tweet engagements and interactions. For the latter, we calculated this by the tweet’s retweet number divided by the user’s followers at the time of posting. In this way, the spread took into account that the number of followers impacts the total number of retweets.

## Results

### Stakeholders / tweeters

**Fig. 2:**
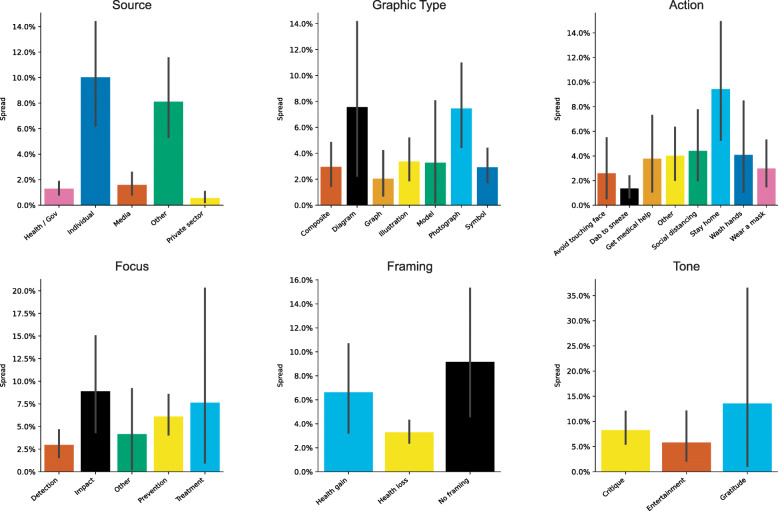
The average spread per category per topic. The bar chart shows the average spread. Colour represents each topic category. The vertical lines depict the confidence intervals (0.95)

The 616 tweets analysed came from 351 verified Twitter accounts. The majority of these users were individuals (*n* = 209, 59.5%), meaning the personal accounts of citizens, activists, politicians, and journalists. This group had the highest spread (10.0%), as indicated by Fig. 2, and accounted for 51.3% of tweets in our sample. The media (*n* = 90, 25.6%) accounted for 27.4% of tweets, and health and government organisations (*n* = 40, 11.3%) had 16.4% of tweets. A possible explanation for the predominance of personal accounts could be that there is a higher fraction of individuals than official organisational accounts on Twitter. Simultaneously, some users authored more than one tweet in the sample (*n* = 89, 25.4%). While the user with the highest number of tweets (*n* = 28) was the WHO, most accounts with multiple tweets belonged to the media (36 accounts) and individuals (35 accounts).

As Table 2 shows, each stakeholder group had a considerable number of followers (the average always in the tens of thousands) and the total number of followers was much higher than the total number of users they themselves were following. This indicates that tweets came mainly from established Twitter accounts who were ‘influencers’. However, each stakeholder group’s average number of followers and their standard deviations indicate a high variability between accounts. This shows that not all the retweeted tweets with images in our sample came from ‘influencers.’

**Table 2:** Per stakeholder summary of the Covid-19 tweets with image dataset

	Total Accounts	Total Following	Total Followers	Average Followers	Standard Deviation Followers	Total Retweets	Average Retweets	Standard Deviation Retweets	Total Tweets	% Sample
Health or Gov	40	33′571	69′946’927	1′748’673	3′571’745	321′076	3179	5′588	101	16.40%
Individuals	209	1′882’736	325′278’634	1′556’357	4′528’980	2′171’560	6872	25′138	316	51.30%
Media	90	182′548	447′279’154	4′969’768	9′902’178	649′205	3841	6′110	169	27.40%
Other	3	11′240	14′098’198	1′566’466	3′049’140	89′488	3729	3′961	24	3.90%
Private Sector	9	641	30′578’973	10′192’991	11′209’315	137′184	22,864	12′602	6	1.00%
Total	351	2′110’736	887′181’886	2′527’584	6′493’767	3′368’513	5468	18′614	616	100%

The tweets came from 35 different countries. The majority of these from the USA (*n* = 267, ~ 43%) and India (*n* = 108, ~ 17.5%). Following were the UK (*n* = 64, ~ 10%), Switzerland (*n* = 32, ~ 5%), Philippines (*n* = 27, ~ 4%), and China (*n* = 21, ~ 3%). Eleven tweets had unknown locations. The fact that other English-speaking regions, notably Australia and New Zealand, were not represented in our sample may be because these regions were less affected by the pandemic.

### Graphic types and visual properties

**Fig. 3:**
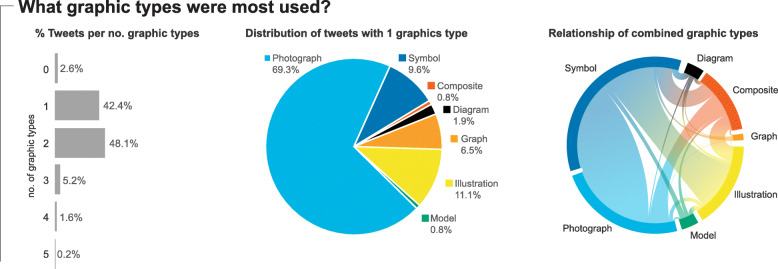
Graphic types used in the most retweeted tweets. The bar chart shows how many graphic types were combined per tweet. The pie chart illustrates how graphic types were used singularly. The chord diagram shows how graphic types were combined

Identified using Saunders typology [22], most tweets (55%) in our sample used a combination of two to five graphic types (see Fig. 3). This was often the case with animated visuals, which accounted for 42.4% (*n* = 261) of all tweets. Figure 3 shows that photographs (either still or moving) were frequently combined with symbols (like institutional logos) (*n* = 177, 44.6%), and symbols were the most combined graphic type overall. This could also have resulted either from branded content or content cross-pollination. For example, media reposted from TikTok or the sharing of WHO branded videos and content.

In tweets with only one graphic type, photographs predominated (*n* = 181), while graphs (n = 17), diagrams (*n* = 5), and models (n = 2) were least used. The fact that 64.4% of tweets (*n* = 397) included in our sample featured photographs and this graphic type had one of the highest spreads (7.5%) highlights their instrumental role as a form of risk communication with great potential to attract attention and be shared. Notably, although diagrams were not frequently used (*n* = 21), this graphic type had the highest spread (7.6%). In 2.6% of tweets, no graphic type was recorded as these tweets used screenshots or text saved in a JPG or PNG format, which did not fit the coding categories [22]. The other characteristics measured showed tweet visuals to be rich in information: 96.9% used colour (*n* = 597), 68.0% (*n* = 419) included text within the image, and 25.8% (*n* = 159) included a URL either in the image or the text. The inclusion of text within the image may indicate that visuals served to compensate for character limitations (Twitter has a limit of 280 characters per Tweet), or alternatively, that visuals helped emphasise messages communicated with text.

### Covid-19 content over time

**Fig. 4:**
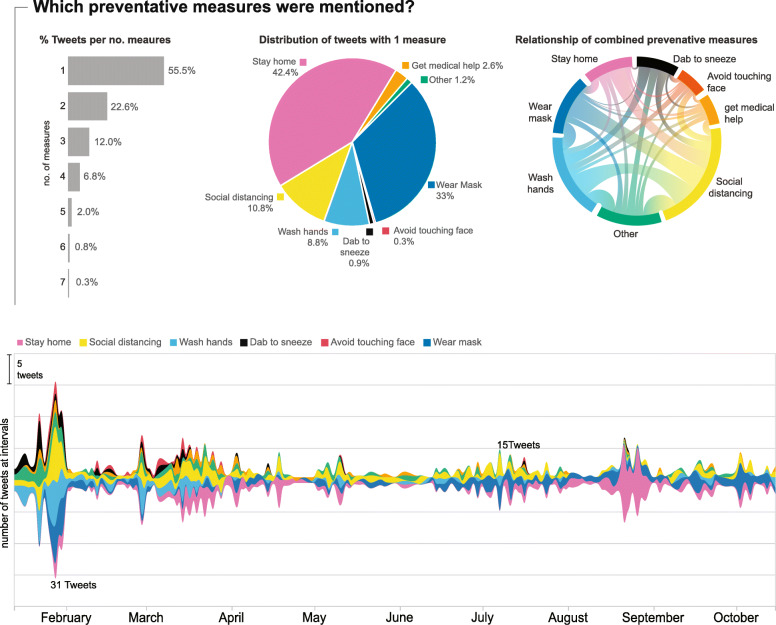
Covid-19 preventative measures communicated and when. The bar chart depicts the number of measures communicated per tweet. The pie chart shows the distribution of measures when communicated singularly. The chord diagram shows how preventative measures were combined. The steam graph shows which measures were communicated and when: set on a central axis, the a-axis shows time and the vertical area represents the number of tweets. Each colour represents a different preventive measure

In complementation to messages of ‘prevention’, our analysis identified the presence of other Covid-19 themes, specifically ‘detection’, ‘treatment’, ‘impact’ and ‘other’. These themes’ average spread is shown in Fig. 2. Overall, Covid-19’s ‘impact’ (tweets communicating how the pandemic was impacting society) was most often present in combination with the topic of ‘detection’ (tweets which referred to the numbers of Covid-19 infections and how to detect the virus from symptoms). Our analysis further revealed that most preventative messages were communicated singularly (55.5%), whereby ‘stay home’ (42.4%) and ‘wear a mask’ (33.0%) frequented most, with the former having the highest average spread overall (9.4%, Fig. 2). A possible explanation for this may be the WHO’s social media #WearAMask challenge and #StayHealthyAtHome challenges. However, when combined (i.e., mentioning two or more preventative measures), as was the case with the remaining 44.5% of the tweets, the preventative measures ‘social distancing’ and ‘wash hands’ frequented more. As an example, one tweet showed citizens at wash stations which were meters apart. The fact that 78.1% of tweets included one or two preventative measures suggests that in our sample less may have been more when it comes to visual risk communication. In other words, tweets containing one or two simple messages may have attracted more attention and thus more retweets, a finding which has important implications for risk communication but needs further research to confirm. Figure 4 presents these results in more detail.

### Risk framing & tone over time

**Fig. 5:**
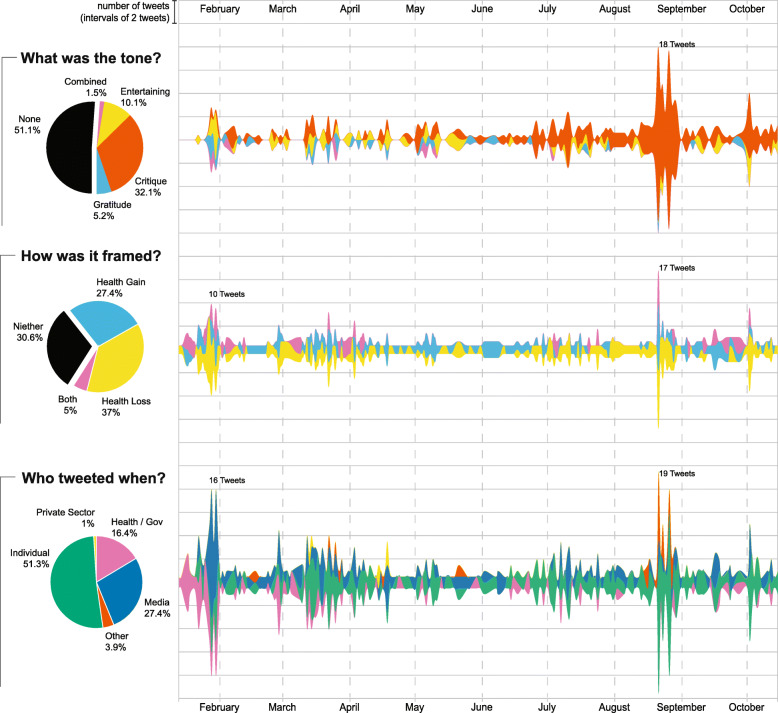
Tone, framing and stakeholders in the most retweeted tweets. The pie graphs depict the percentage of each category. Steam graphs, as stacked area graphs displaced around a central axis, depict the categories present per tweet over time. Each colour represents a different category. The x-axis represents time, the area represents the number of tweets

Of the 616 tweets analysed from January 1 to October 15, 2020, 69.6% used risk framing to communicate preventative measures. Meaning, they either framed messages according to health loss, where the emphasis was on sickness and suffering, or used health gain framing that emphasised protecting and retaining good health. Five percent of tweets used a combination of both. Figure 5 shows that most analysed tweets (37.0%) used health loss framing, particularly around the spikes at the end of January and again in August. A possible explanation is that on January 30th the WHO’s Director-General officially declared Covid-19 as a public health emergency of international concern, their highest level of alarm. The stark rise in cases could have influenced the spike in August (as Covid-19 becomes the third leading cause of death in the US) and an international realisation of the limited beds in intensive care. However, as shown by Fig. 2, the spread of tweets with health gain messages (6.6%) along with tweets with no framing (9.2%) was considerably higher than the spread of tweets with health loss framing (3.3%). This suggests that although health loss framing was more common in the most retweeted tweets, those without framing or with positive framing spread more.

Then in terms of tone, 48.9% (*n* = 301) of tweets were coded showing critique, entertainment or gratitude, and 1.5% of tweets combined tones. Critical tweets were most common overall (*n* = 202, 32.8%) and appeared from June onwards. These were often expressions of disagreement regarding the lack of compliance with preventive measures. For example, critiques of other citizens not wearing masks or of political figures not abiding by regulations. Another example was Indian students protesting against exams as preventative measures could not be followed, and infection could harm families. In contrast, many tweets in our sample around the first half of the year, as shown in Fig. 5, had entertaining tones (overall *n* = 68, 11.0%). These tweets showed, for example, humorous instances of quarantine, like a couple pretending to holiday by fishing on their television screen. Lastly, there were also thankful tweets (*n* = 40, 6.5%) that communicated gratitude for fellow citizens following preventive measures. Interestingly, as shown by Fig. 2, tweets with tones of gratitude had the highest average spread (13.6%).

## Discussion

With 340 million registered users, 166 million daily active users, and 500 million tweets per day [29], Twitter constitutes one of the world’s most widespread communication platforms, especially in a public health crisis. Although social media can help with rapid knowledge dissemination in a pandemic [30], no media is a passive vehicle for communication. Like on other social media platforms, where concise, emotive and immersive content spreads like fire, we have seen Twitter become a “fertile ground for the spread of false information, particularly regarding the ongoing coronavirus disease” [31]. Recognising its role in misinformation propagation, in March 2020, Twitter introduced warning labels for tweets containing potentially harmful or misleading information relating to Covid-19 and linked verified information [32].

Nonetheless, Twitter has played a pivotal part in health risk communication during the Covid-19 health crisis [33–35]. Various world leaders have utilised the platform to inform, boost morale and prompt political discussion [36]. Given such uptake, it is not surprising that health measures trended in the Twittersphere [37–39]. This study documents several health risk measures communicated, often in combination. Most frequently were the measures ‘stay home’ and ‘wear a mask’ — messages focused on actions at the individual level. Concerning the latter, it should be noted that throughout 2020 the WHO iteratively updated their recommendations about mask wearing and even ran social media challenges, such as the #WearAMask and #StayHealthyAtHomes challenges. These updates and efforts could have contributed to the popularity of this topic in the tweets analysed.

Messages targeting individual agency and responsibility for controlling health raise the ethical issue of culpability [40, 41]. As Guttman explains [7], messages that appeal to personal responsibility have pervaded public health communications for decades and can have unintended adverse effects. For example, the tweets shaming citizens for not complying by staying home or wearing a mask could have prompted feelings in non-abiders of guilt, shame or frustration. However, these individuals may not have had a choice, needing to go work to support their family, or not being able to wear a mask for health reasons.

Ethical consideration must also be given to the message framing, specifically regarding the potential for persuasive and paternalistic communication styles, which can create a barrier and lead to erosion of trust. On the other hand, more educational approaches provide only information to enhance rational decision-making, but research shows they are not always effective [42]. In this study, most tweets used health loss or gain framing to persuade adherence to public health measures. Meaning, they presented Covid-19 preventative measures by emphasising their health-protective capacities, or the negative consequences resulting from non-adherence. Out of these two, ‘health gain’ messages spread more (calculated by retweets divided by a user’s number of followers), but ‘health loss’ was the most frequent (in terms of number of tweets in our sample). Appeals to fear using vivid images or describing damages to health echoes earlier public health campaigns, such as smoking or HIV. This approach came under ethical critique for causing unnecessary fear and stigmatisation [7].

However, a public health crisis may justify negative emotional appeals or paternalistic communication strategies to ensure maximal adherence and societal safety [42, 43]. Indeed, prospect theory proposes that loss-framed messages have more success when outcomes are riskier and more uncertain (like in a pandemic with high infectious rates and unclear solutions), while gain-framed messages are more persuasive when outcomes are clearer and more apparent [24, 44–46]. In the Twittersphere, “fear for the unknown nature of the coronavirus” underscored most Covid-19 conversations [37]. This ethical crossroads should be approached with great discretion.

The study results also show that the most retweeted Covid-19 risk communication with visuals took the format of photographs, often with logos and text. One possible reason for this predominance of photos is that they have evidential power by documenting reality. Studies on the role of photographic images also emphasise their multiple roles, including dramatizing experience to increase communicative impact [47]. In other words, they have emotive and rhetorical power and provide easy and quick content for viewers to digest [48–50]. In the context of health communication, research shows visual aids and animated graphics positively influence attention, comprehension, recall and behavioural adherence [51, 52].

Despite the potential of photographs in health communication, some question using their vividness and strong emotional appeal (as common persuasive marketing tactics) to attract attention and convey information about risk [20]. An overtly aesthetic or dramatic approach can force the audience’s attention to particular messages or content to persuade them. However, this may have unintended impacts. For instance, one of the analysed tweets included a video of a conventionally attractive young woman wearing tight clothing and handing out masks to men. Although this video tailored to male viewers successfully drew attention to mask-wearing, it also reinforced negative stereotypes and societal gender/power imbalances.

Still, images transcend literacy and language requirements and so can help promote understanding, accessibility and fairness [7]. Notably, the use of images alongside text is most effective, as was the case in most tweets (68%) in this study. Indeed, combining visual and linguistic signification increases health communication effectiveness [53]. Ultimately, when sensitive to ethical concerns, visual aids can be “among the most highly effective, transparent, fast, memorable, and ethically desirable means of risk communication” [54].

Our analysis of the tweet sources also provides insight into the role of governments and media outlets’ in sharing visual health risk communication on Twitter. Concerning the former, research has shown that a higher intensity of government communication via social media positively influences citizens’ adherence to preventive measures [55]. Previous crisis-related research indicates that health organisations rely more often on traditional media than social media when framing a health crisis [56, 57]. In the context of Covid-19, recent research shows that the tweets of politicians generated the most attention, while those of celebrities attracted the most engagement overall, thereby indicating the value of personal versus official communication channels [58]. Our study results echo this, revealing that individual voices (‘influencers’ with many followers) predominated and had their tweets had the highest spread.

However, health/government institutions and the media also had a significant presence but only at the start. As Fig. 5 illustrates, at the onset of the pandemic, most tweets came from the media (indicated by blue) and health and government organisations (indicated by pink). These stakeholders’ tweets then tapered out into an even distribution. This pattern could reflect citizens’ desire for official guidance at the outset of the pandemic when everything was in a state of uncertainty. The shift towards individual voices from March onwards aligns with the stay-at-home mandates when individual social media use generally increased [39]. The prominence of individual voices highlights the importance of citizens (particularly influencers) sharing health messages among their networks, enabling health messages to reach broader segments of the population and promoting solidarity and inclusiveness.

Finally, the high number of tweets with tones of critique shows how Twitter, even in the context of health-risk communication, gets used as a platform for communicating protest. In September and October, the spikes in critical tweets in our sample came from US tweets, often with political undertones (unsurprising in the lead up to the US election), alongside Indian students protesting against exams. For the latter, authorities were allowing exams despite social distancing and staying home being officially recommended to prevent infections and the spread of Covid-19 [59]. Since the Arab Spring and Occupy Wall Street movements, Twitter has developed a reputation as a platform for protests because it amplifies individual voices, and the mass of critical tweets in this study reflects this. That most tweets used photographs also fits as photos can help build social movements and networks [60, 61], visuals fortify propaganda during conflicts [62] and images can foster advocacy, as we have also seen with climate change movements [63, 64]. Ultimately, this highlights how critical tones about potential damages to health ignite activity on Twitter and that citizens play a crucial role in information distribution.

### Limitations

This study has some limitations. To start, we recognise that filtering for only the top 500 Covid-19 tweets in English per month means the exclusion of other potentially relevant tweets. Moreover, when interpreting our findings, it is important to consider that our analysis presents a snapshot of the retweet counts (as retweet and follower counts were collected at two points in time) and does not account for the potential impact of country-specific events. As well, due to Twitter’s collection limit (a 1% threshold of the entire tweet-volume on Twitter at a given moment) for a short period between mid-March and mid-April the collection using Crowdbreaks was limited to a random subsample of all tweets of interest. However, given the volume of tweets collected the sample is representative and suitable to answer the research questions. This approach’s strength was that it thus revealed the extent to which preventative measures appeared amongst the tweets with the most retweets. However, by only including English language tweets, this study’s results may not reflect global trends as they are biased towards the West. As well, duplicate images were not documented. Another limitation lies in the fact that we limited our analysis to tweets promoting WHO preventative behaviours; this may have led us to miss other types of preventive messages. However, we deemed this a reasonable strategy for verifying the legitimacy and effectiveness of preventive behaviours being promoted on Twitter, as was the study’s focus. Further, although all tweets analysed in this study came from verified accounts, it was beyond this study’s scope to verify Tweet locations’ accuracy and to identify the potential presence of bots.

## Conclusion

To our knowledge, this study is the first to analyse the characteristics, trends, and ethics of Covid-19 visual risk communication on Twitter. This study’s results show that the most retweeted WHO recommended Covid-19 health measures with visuals between January to October 2020 came from personal accounts. This outcome highlights the need for health organisations and governments to engage individuals, particularly influencers, as messengers and advocates, for they enable health messages to reach broader segments of the population, promoting solidarity and inclusiveness.

What characterised the most retweeted tweets with visuals was that they were rich in information: communicating one to two preventive measures, mostly using colour, often animated, and mostly including text and URLs in the image. Further, they often used the visual format of photographs combined with symbols and employed either a health loss or gain framing. Although health gain messages spread more, the predominance of health loss framing combined with photographs as an emotive form raises concerns about persuasive tactics being used to exploit public uncertainty in the midst of a pandemic. However, a public health emergency may justify health and government authorities using such tactics, due to the need for rapid knowledge dissemination and widespread adherence to measures.

Future research is needed to evaluate the behaviour changing efficacy of loss-framed versus gain-framed messages with visuals in the context of the Covid-19 pandemic and across different social media platforms. Future research could also investigate the reasons why these tweets engaged so many Twitter users, specifically to explore if it was due to their content or rather simply because of the influence and number of users followers. As well, research could investigate in more detail how preventative measures spread across different social media platforms and community networks.

## Supplementary Information

**Additional file 1: Tables S1**, **S2**A-F, **S3**.A-C, **S4**.A-B, **S5**.A-D, **S6**.A-B, **S7**.A-B, **S8**. Data showing: tweet locations; spread; graphic types; Covid-19 topics; Covid-19 measures and; tweet framing; tweet tones; stakeholders.

**Additional file 2: Table S9.** Data from qualitative coding. Twitter usernames and all other identifiable information has been removed.

## Data Availability

The full data that support the findings of this study are available from Twitter, but restrictions apply to the availability of these data, which were used under license for the current study, and so are not publicly available. Data are however available from the authors upon reasonable request and with permission of Twitter. The authors declare that all other data supporting the findings of this study, including the identifiers of the analysed tweets, are available within the article and its supplementary information files (see Additional files).

## Abbreviations

WHO: The World Health Organisation
JPEG: Joint Photographic Experts Group
PNG: Portable Network Graphics

## Footnotes

1 Crowdbreaks.org

## References

[1] Herman T. K-pop stars AleXa, Dreamcatcher & IN2IT team up with UNESCO for 'be the future' Covid-19 song. Forbes. 2020. https://www.forbes.com/sites/tamarherman/2020/05/05/k-pop-stars-alexa-dreamcatcher--in2it-team-up-with-unesco-for-be-the-future-covid-19-song/. Accessed 15 Jan 2021.

[2] Seymat T, Gaubert J. Vietnamese COVID-19 video goes viral as prevention message proves popular; EuroNews. 2020. https://www.euronews.com/2020/03/06/coronavirus-vietnamese-covid-19-video-goes-viral-as-prevention-message-proves-popular. Accessed 15 Jan 2021.

[3] Paul K. 'It's corona time': TikTok helps teens cope with coronavirus pandemic. The Guardian. 2020. https://www.theguardian.com/world/2020/mar/12/coronavirus-outbreak-tik-tok-memes. Accessed 15 Jan 2021.

[4] Shinm H (2016). Epidemic and risk communication: an analysis of strategic and graphic characteristics of infographics [dissertation].

[5] Ancker JS, Senathirajah Y, Kukafka R, Starren JB (2006). Design features of graphs in health risk communication: a systematic review. J Am Med Inform Assoc.

[6] Wakefield MA, Loken B, Hornik RC (2010). Use of mass media campaigns to change health behaviour. Lancet.

[7] Guttman N (2017). Ethical issues in health promotion and communication interventions. Oxford research encyclopedia of communication.

[8] Joffe H (2008). The power of visual material: persuasion, emotion and identification. Diogenes..

[9] Avgerinou MD, Pettersson R (2011). Toward a cohesive theory of visual literacy. J Visual Literacy.

[10] Burri RV (2013). Visual power in action: digital images and the shaping of medical practices. Sci Cult.

[11] Armstrong K, Richards AS, Boyd KJ. Red-hot reactance: color cues moderate the freedom threatening characteristics of health PSAs. Health Commun. 2019;36(6):663–70. 10.1080/10410236.2019.1700885. 10.1080/10410236.2019.1700885 31818126

[12] Guidry JPD, Jin Y, Orr CA, Messner M, Meganck S (2017). Ebola on Instagram and twitter: how health organizations address the health crisis in their social media engagement. Public Relat Rev.

[13] Beauchamp DE (1988). The health of the republic: epidemics, medicine, and moralism as challenges to democracy.

[14] Mheidly N, Fares J. Leveraging media and health communication strategies to overcome the COVID-19 infodemic. J Public Health Policy. 2020;41:410–20. 10.1057/s41271-020-00247-w. 10.1057/s41271-020-00247-w PMC7441141 32826935

[15] Castells M (2013). Communication power: OUP Oxford.

[16] Chadwick A. The “social media” maneuver. Soc Media Soc. 2015;1(1):1–2.

[17] Kouzy R, Abi Jaoude J, Kraitem A, El Alam M, Karam B, Adib E (2020). Coronavirus goes viral: quantifying the COVID-19 misinformation epidemic on twitter.

[18] Doan S (2021). Misrepresenting COVID-19: lying with charts during the second Golden age of data design. J Bus Tech Commun.

[19] McIntyre N, Barr C. How UK government misrepresented Covid projections - explained. The Guardian. 2020. https://www.theguardian.com/world/2020/nov/06/how-uk-government-misrepresented-covid-projections-lockdown-explained. Accessed 15 Jan 2021.

[20] Guttman N. Public health communication interventions: values and ethical dilemmas. Sage; 2000.

[21] Lee JL, DeCamp M, Dredze M, Chisolm MS, Berger ZD (2014). What are health-related users tweeting? A qualitative content analysis of health-related users and their messages on twitter. J Med Internet Res.

[22] Saunders A. Graphics and how they communicate. In: Moore D, Dwyer F, editors. Visual literacy: a spectrum of visual learning. Englewood Cliffs: Educational Technology Publications; 1994. p. 183–92.

[23] Organisation WH. WHO COVID-19 transmission and protective measures 2020 [Available from: https://www.who.int/westernpacific/emergencies/covid-19/information/transmission-protective-measures

[24] Tversky A, Kahneman D (1992). Advances in prospect theory: cumulative representation of uncertainty. J Risk Uncertain.

[25] Müller MM, Salathé M (2019). Crowdbreaks: tracking health trends using public social media data and crowdsourcing. Front Public Health.

[26] Cheng Z, Caverlee J, Lee K (2010). You are where you tweet: a content-based approach to geo-locating twitter users. Proceedings of the 19th ACM international conference on information and knowledge management.

[27] Hayes AF, Krippendorff K (2007). Answering the call for a standard reliability measure for coding data. Commun Methods Meas.

[28] Krippendorff K (2011). Agreement and information in the reliability of coding. Commun Methods Meas.

[29] Inc. T. 2020 Twitter announces first quarter 2020 results 2020.

[30] Chan AK, Nickson C, Rudolph J, Lee A, Joynt G. Social media for rapid knowledge dissemination: early experience from the COVID-19 pandemic. Anaesthesia. 2020;75(12):1579–82. 10.1111/anae.15057. 10.1111/anae.15057 PMC7228334 32227594

[31] Al-Rakhami MS, Al-Amri AM (2020). Lies kill, facts save: detecting COVID-19 misinformation in twitter. IEEE Access.

[32] Roy Y, Pickles N. Twitter Blog [Internet]: Twitter. 2020. Available from: https://blog.twitter.com/en_us/topics/product/2020/updating-our-approach-to-misleading-information.html

[33] Raamkumar AS, Tan SG, Wee HL (2020). Measuring the outreach efforts of public health authorities and the public response on Facebook during the COVID-19 pandemic in early 2020: cross-country comparison. J Med Internet Res.

[34] Liao Q, Yuan J, Dong M, Yang L, Fielding R, Lam WWT (2020). Public engagement and government responsiveness in the communications about COVID-19 during the early epidemic stage in China: infodemiology study on social media data. J Med Internet Res.

[35] Dyer J, Kolic B (2020). Public risk perception and emotion on twitter during the Covid-19 pandemic. Appl Netw Sci.

[36] Rufai SR, Bunce C (2020). World leaders’ usage of twitter in response to the COVID-19 pandemic: a content analysis. J Public Health.

[37] Xue J, Chen J, Chen C, Zheng C, Li S, Zhu T. Public discourse and sentiment during the COVID 19 pandemic: using latent Dirichlet allocation for topic modeling on twitter. PLoS One. 2020;15(9):e0239441. 10.1371/journal.pone.0239441. 10.1371/journal.pone.0239441 PMC7518625 32976519

[38] Doogan C, Buntine W, Linger H, Brunt S (2020). Public perceptions and attitudes toward COVID-19 nonpharmaceutical interventions across six countries: a topic modeling analysis of twitter data. J Med Internet Res.

[39] Valdez D, Ten Thij M, Bathina K, Rutter LA, Bollen J (2020). Social media insights into US mental health during the COVID-19 pandemic: longitudinal analysis of twitter data. J Med Internet Res.

[40] Guttman N, Ressler WH. On being responsible: ethical issues in appeals to personal responsibility in health campaigns. J Health Commun. 2001;6(2):117–36. 10.1080/10810730116864. 10.1080/108107301750254466 11405077

[41] Coleman R, Hatley ML (2014). Ethical health communication: a content analysis of predominant frames and primes in public service announcements. J Mass Media Ethics.

[42] Bester JC (2015). Vaccine refusal and trust: the trouble with coercion and education and suggestions for a cure. J Bioethical Inquiry.

[43] Resnik DB (2001). Ethical dilemmas in communicating medical information to the public. Health Policy.

[44] Toll BA, O'Malley SS, Katulak NA, Wu R, Dubin JA, Latimer A (2007). Comparing gain-and loss-framed messages for smoking cessation with sustained-release bupropion: a randomized controlled trial. Psychol Addict Behav.

[45] Ruggeri K, Alí S, Berge ML, Bertoldo G, Bjørndal LD, Cortijos-Bernabeu A (2020). Replicating patterns of prospect theory for decision under risk. Nat Hum Behav.

[46] Abood DA, Coster DC, Mullis AK, Black DR (2002). Evaluation of a “loss-framed” minimal intervention to increase mammography utilization among medically un-and under-insured women. Cancer Detect Prev.

[47] Hand M. Ubiquitous photography. Cambridge, UK and Malden: Polity; 2012.

[48] McCloud S (1994). Understanding comics: the invisible art.

[49] Goldstein CS. Capturing the German eye: American visual propaganda in occupied Germany. Chicago: University of Chicago Press; 2009. 10.7208/chicago/9780226301716.001.0001.

[50] Seo H, Kinsey DF (2012). Meaning of democracy around the world: a thematic and structural analysis of videos defining democracy. Vis Commun Q.

[51] King AJ. A content analysis of visual cancer information: prevalence and use of photographs and illustrations in printed health materials. Health Commun. 2015;30(7):722–31. 10.1080/10410236.2013.878778. 10.1080/10410236.2013.878778 25061954

[52] Sontag JM, Barnes SR (2017). The visual framing of graphics when used in preventative health digital news packages: exploring the use of a narrative structure as the message infrastructure. J Visual Commun Med.

[53] Houts PS, Doak CC, Doak LG, Loscalzo MJ (2006). The role of pictures in improving health communication: a review of research on attention, comprehension, recall, and adherence. Patient Educ Couns.

[54] Garcia-Retamero R, Cokely ET (2011). Effective communication of risks to young adults: using message framing and visual aids to increase condom use and STD screening. J Exp Psychol Appl.

[55] Al-Hasan A, Yim D, Khuntia J (2020). Citizens’ adherence to COVID-19 mitigation recommendations by the government: a 3-country comparative evaluation using web-based cross-sectional survey data. J Med Internet Res.

[56] Liu BF, Kim S (2011). How organizations framed the 2009 H1N1 pandemic via social and traditional media: implications for US health communicators. Public Relat Rev.

[57] Alonso-Cañadas J, Galán-Valdivieso F, Saraite-Sariene L, Caba-Pérez C (2020). Committed to health: key factors to improve users’ online engagement through Facebook. Int J Environ Res Public Health.

[58] Kamiński M, Szymańska C, Nowak JK (2021). Whose tweets on COVID-19 gain the Most attention: celebrities, political, or scientific authorities?. Cyberpsychol Behav Soc Netw.

[59] Roy B, Roy A (2020). Conducting examinations in India: emergency, contention and challenges of students amidst covid-19 pandemic. Child Youth Serv Rev.

[60] Gaby S, Caren N (2012). Occupy online: how cute old men and Malcolm X recruited 400,000 US users to OWS on Facebook. Soc Mov Stud.

[61] Akdag Salah AA, Scharnhorst A, Ten Bosch O, Doorn P, Manovich L, Salah AA (2012). Significance of visual interfaces in institutional and user-generated databases with category structures. Proceedings of the second international ACM workshop on personalized access to cultural heritage.

[62] Seo H (2014). Visual propaganda in the age of social media: an empirical analysis of twitter images during the 2012 Israeli–Hamas conflict. Vis Commun Q.

[63] Hopke JE, Hestres LE. Visualizing the Paris climate talks on twitter: media and climate stakeholder visual social media during COP21. Soc Media Soc. 2018;4(3):1–15. 10.1177/2056305118782687.

[64] Lehman B, Thompson J, Davis S, Carlson JM (2019). Affective images of climate change. Front Psychol.

