# Supplementary material for: Qualitative analysis of visual risk communication on twitter during the Covid-19 pandemic
Source: BMC Public Health. 2021 Apr 28;21:810. doi: 10.1186/s12889-021-10851-4 (PMC8079223; doi:10.1186/s12889-021-10851-4)
Supplement: Supplementary file 1 — Additional file 1: Tables S1, S2A-F, S3.A-C, S4.A-B, S5.A-D, S6.A-B, S7.A-B, S8. Data showing: tweet locations; spread; graphic types; Covid-19 topics; Covid-19 measures and; tweet framing; tweet tones; stakeholders. [file 12889_2021_10851_MOESM1_ESM.pdf]

**Qualitative Analysis of Visual Risk Communication on Twitter during the Covid-19 Pandemic**

Joanna Sleigh, Julia Amann, Manuel Schneider, Effy Vayena

# Additional file 1

**Table S1.** Summary of Tweet Locations

| **Countries** | **Total** |
|---|---|
| United States of America | 268 |
| India | 108 |
| United Kingdom | 64 |
| Switzerland | 32 |
| Philippines | 27 |
| China | 21 |
| (Unknown) | 11 |
| South Korea | 10 |
| Nigeria | 8 |
| Malaysia | 7 |
| Ireland | 6 |
| Russia | 6 |
| Australia | 6 |
| Thailand | 5 |
| South Africa | 4 |
| New Zealand | 4 |
| Canada | 4 |
| Qatar | 4 |
| France | 3 |
| Japan | 2 |
| Singapore | 2 |
| Germany | 1 |
| Jordan | 1 |
| Uganda | 1 |
| Italy | 1 |
| Belgium | 1 |
| Rwanda | 1 |
| Indonesia | 1 |
| Saudi Arabia | 1 |
| United Arab Emirates | 1 |
| Norway | 1 |
| Kenya | 1 |
| Maldives | 1 |
| Israel | 1 |
| Republic of Congo | 1 |

**Qualitative Analysis of Visual Risk Communication on Twitter during the Covid-19 Pandemic**

Joanna Sleigh, Julia Amann, Manuel Schneider, Effy Vayena

**Table S2.A** Spread per Stakeholder / Source

| | Health or Gov | Media | Other | Individual | Private Sector |
|---|---|---|---|---|---|
| **count** | 101 | 169 | 24 | 316 | 6 |
| **mean** | 1.29% | 1.59% | 8.12% | 10.02% | 0.55% |
| **std** | 2.76% | 5.83% | 8.05% | 37.06% | 0.59% |

**Table S2.B** Spread per Graphic Type

| | Composite | Diagram | Graph | Illustrations | Model | Photograph | Symbol |
|---|---|---|---|---|---|---|---|
| **count** | 78 | 21 | 28 | 139 | 16 | 397 | 316 |
| **mean** | 2.96% | 7.56% | 2.04% | 3.37% | 3.28% | 7.46% | 2.93% |
| **std** | 7.69% | 13.91% | 5.06% | 9.83% | 8.39% | 33.01% | 11.8% |

**Table S2.C** Spread per Covid-19 Action

| | Avoid touching face | Dab to sneeze | Get medical help | Social Distancing | Other | Wash hands | Wear Masks | Stay Home |
|---|---|---|---|---|---|---|---|---|
| **count** | 29 | 48 | 55 | 237 | 104 | 150 | 245 | 246 |
| **mean** | 2.59% | 1.35% | 3.78% | 4.41% | 4.02% | 4.09% | 2.99% | 9.44% |
| **std** | 7.3% | 3.28% | 12.37% | 22.87% | 11.47% | 24.17% | 16.05% | 37.36% |

**Table S2.D** Spread per Covid-19 Focus

| | Detection | Impact | Other | Prevention | Treatment |
|---|---|---|---|---|---|
| **count** | 145 | 170 | 7 | 616 | 45 |
| **mean** | 2.96% | 8.89% | 4.16% | 6.11% | 7.63% |
| **std** | 9.17% | 38.96% | 6.84% | 27.09% | 38.69% |

**Table S2.E** Spread per Message Framing

| | Health Gain | Health Loss | Non Applicable |
|---|---|---|---|
| **count** | 200 | 260 | 187 |
| **mean** | 6.62% | 3.29% | 9.16% |
| **std** | 28.32% | 8.06% | 38.41% |

**Table S2.F** Spread per Message Tone

| | Entertainment | Criticism | Gratitude |
|---|---|---|---|
| **count** | 68 | 202 | 40 |
| **mean** | 5.81% | 8.26% | 13.56% |
| **std** | 20.83% | 23.83% | 67.49% |

**Qualitative Analysis of Visual Risk Communication on Twitter during the Covid-19 Pandemic**

Joanna Sleigh, Julia Amann, Manuel Schneider, Effy Vayena

## **Table S3.A** Graphic types communicated individually

| What | Total | Percent |
|---|---|---|
| Composite | 2 | 0.80% |
| Diagram | 5 | 1.90% |
| Graph | 17 | 6.50% |
| Illustrations | 29 | 11.10% |
| Models | 2 | 0.80% |
| Photograph | 181 | 69.30% |
| Symbol | 25 | 9.60% |

## **Table S3.B** Graphic types combined

| What | Total | Percent |
|---|---|---|
| Zero graphic types | 16 | 2.60% |
| One graphic type | 261 | 42.40% |
| two graphic types | 296 | 48.10% |
| three graphic types | 32 | 5.20% |
| four graphic types | 10 | 1.60% |
| five graphic types | 1 | 0.20% |

## **Table S3.C** Normed edges of combined graphic types

| From | To | Weight |
|---|---|---|
| Diagram: process, flow of results... | Composite Graphics (multiple-images) | 0.01511879 |
| Graph: show quantitative relationships | Composite Graphics (multiple-images) | 0.00647948 |
| Graph: show quantitative relationships | Diagram: process, flow of results... | 0.00215983 |
| Illustrations or rendered pictures | Composite Graphics (multiple-images) | 0.06479482 |
| Illustrations or rendered pictures | Diagram: process, flow of results... | 0.01511879 |
| Illustrations or rendered pictures | Graph: show quantitative relationships | 0.00215983 |
| Models (such as 3d) | Composite Graphics (multiple-images) | 0.01727862 |
| Models (such as 3d) | Diagram: process, flow of results... | 0 |
| Models (such as 3d) | Graph: show quantitative relationships | 0 |
| Models (such as 3d) | Illustrations or rendered pictures | 0.01943844 |
| Photograph | Composite Graphics (multiple-images) | 0.08855292 |
| Photograph | Diagram: process, flow of results... | 0.00647948 |
| Photograph | Graph: show quantitative relationships | 0.00215983 |
| Photograph | Illustrations or rendered pictures | 0.01511879 |
| Photograph | Models (such as 3d) | 0.00647948 |
| Symbol: pictographic or abstract | Composite Graphics (multiple-images) | 0.06911447 |
| Symbol: pictographic or abstract | Diagram: process, flow of results... | 0.02807775 |
| Symbol: pictographic or abstract | Graph: show quantitative relationships | 0.01511879 |
| Symbol: pictographic or abstract | Illustrations or rendered pictures | 0.21814255 |
| Symbol: pictographic or abstract | Models (such as 3d) | 0.02591793 |
| Symbol: pictographic or abstract | Photograph | 0.38228942 |

**Qualitative Analysis of Visual Risk Communication on Twitter during the Covid-19 Pandemic**

Joanna Sleigh, Julia Amann, Manuel Schneider, Effy Vayena

**Table S4.A** Covid-19 topics communicated individually, aside from prevention

| What | Total |
|---|---|
| Detection | 45 |
| Impact | 170 |
| Other | 0 |
| Treatment | 2 |
| None | 341 |

**Table S4.B** Covid-19 topics combined

| What | Total |
|---|---|
| One topic | 341 |
| Two topics | 205 |
| Three topics | 48 |
| Four topics | 22 |

**Table S5.A** Covid-19 measures communicated individually

| Intent | Sum | Total |
|---|---|---|
| Wear a mask | 113 | 33.00% |
| Avoid touching | 1 | 0.30% |
| sneezing | 3 | 0.90% |
| Wash hands | 30 | 8.80% |
| Social distance | 37 | 10.80% |
| Stay home | 145 | 42.40% |
| Get medical help | 9 | 2.60% |
| Other | 4 | 1.20% |

**Table S5.B** Overall frequency of Covid-19 measures

| What | Total |
|---|---|
| Wear a mask | 245 |
| Avoid touching | 29 |
| sneezing | 48 |
| Wash hands | 150 |
| Social distance | 237 |
| Stay home | 246 |
| Get medical help | 55 |
| Other | 104 |

**Table S5.C** Covid-19 measures combined

| Number combined | Total | Percent |
|---|---|---|
| one | 342 | 55.50% |
| two | 139 | 22.60% |
| three | 74 | 12.00% |
| four | 42 | 6.80% |

| | | |
|---|---|---|
| five | 12 | 1.90% |
| six | 5 | 0.80% |
| seven | 2 | 0.30% |

## **Table S5.D** Normed edges of combined Covid-19 measures

| From | To | Weight |
|---|---|---|
| Cover mouth & nose when sneezing | Avoid touching mouth and eyes | 0.02 |
| Get medical help w. symptoms (call - don't go in) | Avoid touching mouth and eyes | 0.01294118 |
| Get medical help w. symptoms (call - don't go in) | Cover mouth & nose when sneezing | 0.00823529 |
| Keep distance | Avoid touching mouth and eyes | 0.02941176 |
| Keep distance | Cover mouth & nose when sneezing | 0.04588235 |
| Keep distance | Get medical help w. symptoms (call - don't go in) | 0.02941176 |
| Other | Avoid touching mouth and eyes | 0.01647059 |
| Other | Cover mouth & nose when sneezing | 0.04 |
| Other | Get medical help w. symptoms (call - don't go in) | 0.02470588 |
| Other | Keep distance | 0.07058824 |
| Wash hands | Avoid touching mouth and eyes | 0.03176471 |
| Wash hands | Cover mouth & nose when sneezing | 0.05294118 |
| Wash hands | Get medical help w. symptoms (call - don't go in) | 0.01882353 |
| Wash hands | Keep distance | 0.11176471 |
| Wash hands | Other | 0.06235294 |
| Wear a mask | Avoid touching mouth and eyes | 0.00470588 |
| Wear a mask | Cover mouth & nose when sneezing | 0.00352941 |
| Wear a mask | Get medical help w. symptoms (call - don't go in) | 0.01411765 |
| Wear a mask | Keep distance | 0.10588235 |
| Wear a mask | Other | 0.03294118 |
| Wear a mask | Wash hands | 0.05529412 |
| Work from home | Avoid touching mouth and eyes | 0.00705882 |
| Work from home | Cover mouth & nose when sneezing | 0.00823529 |
| Work from home | Get medical help w. symptoms (call - don't go in) | 0.01882353 |
| Work from home | Keep distance | 0.08235294 |
| Work from home | Other | 0.02588235 |
| Work from home | Wash hands | 0.02941176 |
| Work from home | Wear a mask | 0.03647059 |

## **Table S6.A** Number of tweets with framing

| Framing | Total | Percent |
|---|---|---|
| Health Gain | 169 | 27.40% |
| Health Loss | 229 | 37% |
| Both Gain & Loss | 31 | 5% |
| Neither | 187 | 30.60% |

## **Table S6.B** Framing used from January until October 2020

| Month | Only Health Gain | Only Health Loss | Both Gain & Loss | Neither | Total |
|---|---|---|---|---|---|
| Jan | 20 | 36 | 7 | 27 | 90 |
| Feb | 11 | 15 | 3 | 19 | 48 |

| | | | | | |
|---|---|---|---|---|---|
| Mar | 29 | 29 | 4 | 41 | 103 |
| Apr | 18 | 20 | 4 | 12 | 54 |
| May | 18 | 10 | 0 | 8 | 36 |
| Jun | 16 | 9 | 0 | 6 | 31 |
| Jul | 12 | 34 | 4 | 24 | 74 |
| Aug | 19 | 46 | 3 | 37 | 105 |
| Sep | 19 | 5 | 5 | 7 | 36 |
| Oct | 6 | 25 | 1 | 7 | 39 |

**Table S7.A** Different tones present in the Tweets

| What | Total | Percent |
|---|---|---|
| Combination | 9 | 1.50% |
| Criticism | 198 | 32.10% |
| Entertainment | 62 | 10.10% |
| Gratitude | 32 | 5.20% |
| None | 315 | 51.10% |

**Table S7.B** Tones from January until October 2020

| Month | Combination | Criticism | Entertainment | Gratitude | None | Total |
|---|---|---|---|---|---|---|
| Jan | 2 | 2 | 8 | 6 | 72 | 90 |
| Feb | 1 | 7 | 4 | 3 | 33 | 48 |
| Mar | 2 | 11 | 11 | 7 | 72 | 103 |
| Apr | 0 | 5 | 9 | 4 | 36 | 54 |
| May | 3 | 9 | 8 | 4 | 12 | 36 |
| Jun | 0 | 11 | 1 | 2 | 17 | 31 |
| Jul | 1 | 32 | 9 | 2 | 30 | 74 |
| Aug | 0 | 87 | 4 | 1 | 13 | 105 |
| Sep | 0 | 12 | 4 | 2 | 18 | 36 |
| Oct | 0 | 22 | 5 | 0 | 12 | 39 |

**Table S8.** Stakeholder presence from January until October 2020

| Month | Health / Gov | Individual | Media | Other | Private Sector | Total |
|---|---|---|---|---|---|---|
| Jan | 37 | 17 | 36 | 0 | 0 | 90 |
| Feb | 13 | 15 | 19 | 1 | 0 | 48 |
| Mar | 17 | 53 | 28 | 3 | 2 | 103 |
| Apr | 5 | 26 | 18 | 1 | 4 | 54 |
| May | 4 | 20 | 11 | 1 | 0 | 36 |
| Jun | 6 | 21 | 4 | 0 | 0 | 31 |
| Jul | 7 | 46 | 21 | 0 | 0 | 74 |
| Aug | 4 | 68 | 15 | 18 | 0 | 105 |
| Sep | 5 | 22 | 9 | 0 | 0 | 36 |
| Oct | 3 | 28 | 8 | 0 | 0 | 39 |
